# Analysis of Minerals as Electrode Materials for Ca-based Rechargeable Batteries

**DOI:** 10.1038/s41598-019-46002-4

**Published:** 2019-07-04

**Authors:** A. Torres, F. J. Luque, J. Tortajada, M. E. Arroyo-de Dompablo

**Affiliations:** 1 Departamento de Química Inorgánica, Facultad de Cc. Químicas, Universidad Complutense de Madrid, 28040 Madrid, Spain; 2 Departamento de Mineralogía y Petrología, Facultad de Geología, Universidad Complutense de Madrid, 28040 Madrid, Spain; 3 Departamento Geomateriales, Instituto de Geociencias IGEO (CSIC, UCM), 28040 Madrid, Spain; 4 Departamento de Química Física, Facultad de Cc. Químicas, Universidad Complutense de Madrid, 28040 Madrid, Spain

**Keywords:** Batteries, Batteries, Geochemistry

## Abstract

Rechargeable lithium-ion batteries dominate the consumer electronics and electric vehicle markets. However, concerns on Li availability have prompted the development of alternative high energy density electrochemical energy storage systems. Rechargeable batteries based on a Ca metal anode can exhibit advantages in terms of energy density, safety and cost. The development of rechargeable Ca metal batteries requires the identification of suitable high specific energy cathode materials. This work focuses on Ca-bearing minerals because they represent stable and abundant compounds. Suitable minerals should contain a transition metal able of being reversibly reduced and oxidized, which points to several major classes of silicates and carbonates: olivine (CaFeSiO_4_; kirschsteinite), pyroxene (CaFe/MnSi_2_O_6_; hedenbergite and johannsenite, respectively), garnet (Ca_3_Fe/Cr_2_Si_3_O_12_; andradite and uvarovite, respectively), amphibole (Ca_2_Fe_5_Si_8_O_22_(OH)_2_; ferroactinolite) and double carbonates (CaMn(CO_3_)_2_; kutnahorite and CaFe(CO_3_)_2_; ankerite). This work discusses their electrode characteristics based on crystal chemistry analysis and density functional theory (DFT) calculations. The results indicate that upon Ca deintercalation, compounds such as pyroxene, garnet and double carbonate minerals could display high theoretical energy densities (ranging from 780 to 1500 Wh/kg) with moderate structural modifications. As a downside, DFT calculations indicate a hampered Ca mobility in their crystal structures. The overall analysis then disregards olivine, garnet, pyroxene, amphibole and double carbonates as structural types for future Ca-cathode materials design.

## Introduction

**Figure 1:**
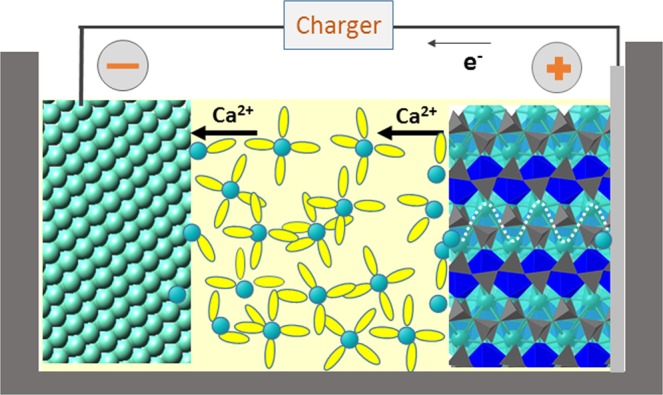
Scheme of a Ca-based battery using pyroxene as the positive electrode and Ca metal as the negative electrode. During the charge of the battery Ca ion are deinserted from the positive electrode and deposited in the Ca metal, and the electrons flow across the external circuit.

While the current state-of-the-art in rechargeable batteries is the Li-ion technology, research efforts are being intensified towards the development of alternative technologies to satisfy the ever-increasing demand for enhanced energy density and lower costs. This prompted the development of rechargeable batteries based on the more abundant and cheaper Ca, Mg and Al metals^1,2^. Like in the Li-ion technology, the operation of Ca or Mg batteries relies on a reversible intercalation reaction (see Fig. 1). During the discharge of the electrochemical cell, the mobile ion (Ca^2+^/Mg^2+^) moves from the metal anode and intercalates in the positive electrode, while the electrons flow across an external circuit (reduction of the cathode material). When charging the battery, the reactions are reversed as shown in Fig. 1. The energy density delivered by the electrochemical cell depends on both the electrochemical capacity and the operation potential. The capacity is governed by the ratio between the difference in inserted ion contents in the intercalated and deintercalated states of the positive electrode, and its overall formula weight. The operation potential depends on the nature of the redox centers (the intercalant ion and a transition metal element in the positive electrode). Batteries based on divalent charge carriers (Ca^2+^, Mg^2+^) can exhibit advantages in terms of energy density since for a certain amount of intercalated ions the expected capacity is doubled when compared to single valent carriers like Li^+^ or Na^+^. In addition, Ca offers a low reduction potential (only 0.3 V below Li), so operation voltages are similar in the Li and Ca intercalation chemistries.

Under the perspective of high specific energies (exceeding 600 Wh/kg positive electrode material), researchers from various disciplines have joined endeavors in the quest of suitable electrode and electrolytes for the emerging Ca technology. Although some promising results have been achieved, the identification of high specific energy electrode materials remains a challenge^3–9^. Because minerals represent stable compounds in natural conditions, in this work we discuss the possible applications of Ca-bearing minerals as electrode materials for Ca-based batteries.

Calcium is the fifth element in abundance in the Earth’s crust, representing around 3.65 wt.%^10^. This is therefore one of its main advantages respect to other elements (mainly Li) used in battery technology. In addition to plagioclase feldspars (Na_1−0_Ca_0−1_Al_1−2_Si_3−2_ O_8_), the most abundant calcium minerals are carbonates (calcite and aragonite, CaCO_3_; dolomite, CaMg(CO_3_)_2_), that constitute about 2.5% of the crust^11^, and sulfates (gypsum, CaSO_4_ 2H_2_O; anhydrite, CaSO_4_). All these industrial minerals can be also used to obtain metallic calcium^12^ which eventually forms the anode in the battery. The most common industrial procedure to obtain Ca metal is to treat the mineral with HCl to form CaCl_2_ and a subsequent elecytrolisis process^13–15^. In addition, calcium metal is produced by an aluminothermic reduction process that begins with CaCO_3_ calcined to form CaO^14,16,17^. It has been recently reported^18^ that for a Li-ion battery the cost of elemental lithium (considering both the electrolyte and electrodes) is about 44 USD per kilowatt hour. This calculation was made according to the price of Li_2_CO_3_ in 2015 (about 6.5 USD/kg). The final price in the market for Ca metal in 2014 was about 4.3 USD/kg^14^. The current price of battery-grade Li_2_CO_3_ is close to 15 USD/kg, therefore much higher than the price of Ca metal. This supports the expected lower cost of the Ca-battery technology, assuming that all other components are equally affordable for both technologies. Additionally, the positive electrode material used in the highest energy density commercial Li-ion batteries contains nickel and cobalt (scarce metals), and this represents the major bottleneck for cost reduction. Reaching high energy densities utilizing Ni and Co free positive electrode materials is a key viewpoint in the calcium technology.

Minerals with potential application as positive electrode in the Ca-battery technology should contain a transition metal able of being reversibly reduced (cathode during the discharge of the cell) and oxidized (anode during the charge of the cell). Minerals containing both Ca and transition metals are by far less abundant than the above mentioned Ca-bearing minerals, and belong to the major classes of silicates and carbonates^19^. Due to size effects, Ca usually combines with Fe and Mn, and more rarely with Cr. Among the silicates, the interesting minerals would be within the olivine (CaFeSiO_4_; kirschsteinite), pyroxene (CaFe/MnSi_2_O_6_; hedenbergite and johannsenite, respectively), garnet (Ca_3_Fe/Cr_2_Si_3_O_12_; andradite and uvarovite, respectively), and amphibole (Ca_2_Fe_5_Si_8_O_22_(OH)_2_; ferroactinolite) groups. In addition to silicates, other minerals containing Ca and Mn/Fe are the dolomite-like structural counterparts kutnahorite (CaMn(CO_3_)_2_) and ankerite (CaFe(CO_3_)_2_). Along with intercalation voltage and specific capacity (which determine the specific energy), a good ionic diffusion (D ~ 10^−12^ cm^2^ s^−1^) is a prerequisite for an electrode material. Indeed, one of the major concerns in the cathode design for the divalent battery technology is the limited mobility of Mg^2+^ and Ca^2+^ ions in inorganic structures. The migration energy barriers, which can be extracted from DFT calculations, provide an approximate estimate of the ionic diffusivity^20^. It has been estimated that for a reasonable cell power rate (discharging time 2 hours), the energy barriers for cation diffusion should be below 0.525 eV in micrometer particles and 0.625 eV in nanosized particles^3^. Previous computational works found appealing calculated energy barriers of 0.7 eV for some virtual CaM_2_O_4_ spinel with Ca occupying the tetrahedral sites^4^. Energy barriers above 1 eV are reported for perovskite and post-spinel transition metal oxides that actually resulted inactive as positive electrode in Ca cells^21,22^. Aiming to explore the potential of minerals as electrode material for Ca-batteries, after a rational pre-evaluation, we use DFT calculations to anticipate the relevant electrochemical characteristics of pyroxene, garnets and double carbonates.

## Results

A simple analysis of chemical formulae and crystal structure serves to discard those minerals with low theoretical specific capacities or no pathways for Ca diffusion. So, a low specific capacity is expected for minerals of the amphibole group. The deintercalation of all Ca ions from this mineral structure would oxidize the Fe^2+^ ions to the formal oxidation state Fe^+2.8^, according to the reaction:

1 $${{\rm{Ca}}}_{{\rm{2}}}{{\rm{Fe}}}_{{\rm{5}}}{{\rm{Si}}}_{{\rm{8}}}{{\rm{O}}}_{{\rm{22}}}{({\rm{OH}})}_{{\rm{2}}}\to {\rm{2}}\,{\rm{Ca}}+{{\rm{Fe}}}_{{\rm{5}}}{{\rm{Si}}}_{{\rm{8}}}{{\rm{O}}}_{{\rm{22}}}{({\rm{OH}})}_{{\rm{2}}}$$

The expression for the maximum specific capacity delivered by the positive electrode material, can be written as:

2 $${C}_{s}(\frac{mAh}{{g}})=\frac{zyF}{3.6Wt}$$

where z is the charge of the intercalant ion (for Ca, z = 2), y the amount of inserted Ca (y = 2 for amphibole) and W_t_ is the formula weight of the mineral (801.6 g/mol in this case) Thus, the maximum theoretical capacity of Ca_2_Fe_5_Si_8_O_22_(OH)_2_ is 133.7 mAh/g, by far too low for practical applications.

**Figure 2:**
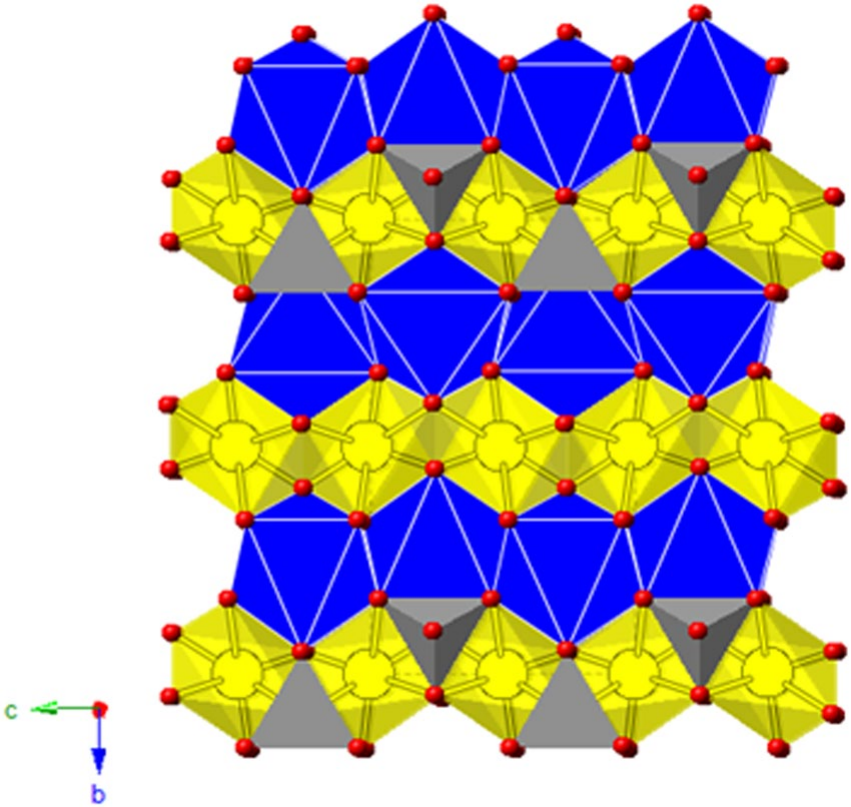
View of the olivine structure showing the octahedral M1 (in yellow) and M2 (in blue) sites, and tetrahedral Si (in grey). The M1 sites form channels for cation diffusion.

Olivine-CaFeSiO_4_ would deliver a maximum theoretical capacity of 285 mAh/g upon Ca deintercalation (redox couple Fe^2+^/Fe^4+^). Noteworthy, olivine-type LiFePO_4_ is currently used as positive electrode in commercial Li-ion batteries, and MgMSiO_4_ (M = Fe, Mn) have been proposed as positive electrode for Mg batteries^20,23–25^. Figure 2 shows the crystal structure of olivine. It is usually described in terms of a hexagonal close-packing of oxygen with Mg and transition metal (Fe, Mn) ions located in half of the octahedral sites, and Si in one eight of the tetrahedral positions. The octahedral sites are further of two types, namely, M1 and M2. The M1 octahedron geometry is comparatively more distorted and smaller, whereas M2 octahedron is more regular and larger^26^. The M2 octahedra form a network of corner-sharing octahedra with low connectivity (in blue in Fig. 2). The M1 octahedra form edge-shared chains along the *c*-axis, providing a channel for cation diffusion (in yellow in Fig. 2). Contrary to the smaller Li and Mg ions, the Ca ions in kirschsteinite (CaFeSiO_4_) occupy the M2 octahedral site^26,27^. With no diffusion pathways permitting Ca mobility, Ca-based olivines were discarded as electrode materials.

As detailed below, pyroxene, garnet and double carbonates have pathways for Ca diffusion and theoretical capacities exceeding 200 mAh/g. DFT provides a good description of their crystal structures with the differences between calculated and experimental lattice parameters being below 5% (see Table 1 in S.I.). The results for each mineral group are discussed below.

### Pyroxene group

**Figure 3:**
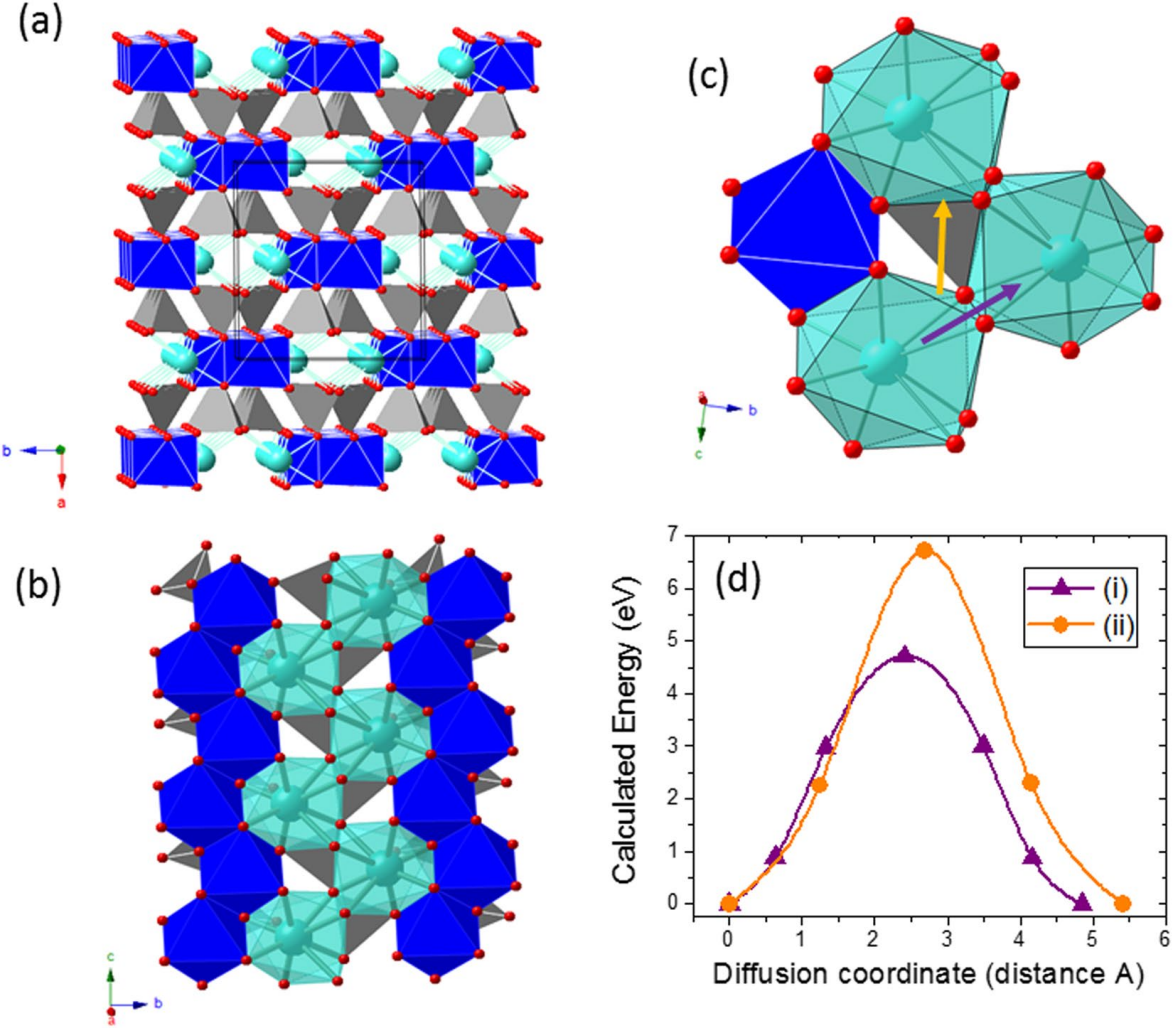
Analysis of the pyroxene minerals. (**a**,**b**) Crystal structure on (001) showing the chain arrangement and location of M1 sites (in dark blue) and M2 sites (in cyan). Note that both sites form rows of edge-sharing polyhedra along the c-axis. (**c**,**d**) Two possible pathways for Ca diffusion along the *c* axis in pyroxene CaMn(SiO_3_)_2_ and their corresponding calculated energy barriers. Color code: Ca cyan, Mn blue, Si grey, O red.

Most common natural pyroxenes are Fe/Mg-bearing silicates, with the general formula XYZ_2_O_6_. The Z cations correspond to Si^4+^, with very minor substitution for Al^3+^. Although pyroxene composition is quite variable (see Table 2 in S.I.), the most usual cations in natural pyroxenes are X = Mg^2+^, Fe^2+^, Ca^2+^, Na^+^ and Y = Mg^2+^, Fe^2+^, Al^3+^ ^28,29^. The crystal structure of pyroxenes (Fig. 3) is characterized by single chains of [SiO_4_]^4−^ tetrahedra extending along the *c*-axis. In these chains, each tetrahedron shares two oxygens with their neighbours. As seen in the (001) projection of the structure (Fig. 3a), the chains are stacked atop each other in the *a*-axis direction in an alternating fashion, leading to two different sites: those located between the bases of tetrahedra (the M2 sites, in cyan) which contain the X cations in distorted 6-fold or 8-fold coordination, and those between unshared apical oxygens (the M1 octahedral sites occupied by the Y cations, in blue). The coordination of the M2 sites depends on how the chains are stacked and which cation occupies the site. When the cation is relatively small (e.g., Mg^2+^ or Fe^2+^) the M2 site has octahedral coordination and the whole structure has orthorhombic symmetry (so, they are usually called orthopyroxenes). For larger cations (e. g., Ca^2+^, Na^+^) its coordination is 8-fold and the structure of the pyroxenes is monoclinic (clinopyroxenes). The M2 sites are interconnected forming a zig-zag channel for cation diffusion (Fig. 3b). Besides Fe, other transition metals (Mn, Ti) may occur in the M1 sites of natural pyroxenes in minor amounts. In addition, there are many reports on the preparation of a variety of synthetic pyroxenes with the general formula CaM(SiO_3_)_2_ (M = transition metal ion)^30–33^.

The maximum theoretical capacity of CaM(SiO_3_)_2_ pyroxenes is around 215 mAh/g, following the full Ca deintercalation and concomitant oxidation of M^2+^ to M^4+^. The average intercalation voltages of the Ca deintercalation reaction can be obtained from the calculated total energy of the initial (CaM(SiO_3_)_2_ and deintercalated (M(SiO_3_)_2_) forms^34^, according with equation (3):

3 $$V(V)=\frac{-{E}_{TOTAL}(Initial)+{E}_{TOTAL}(De{int}erclated)+y{E}_{TOTAL}(Ca)}{2F}$$

**Figure 4:**
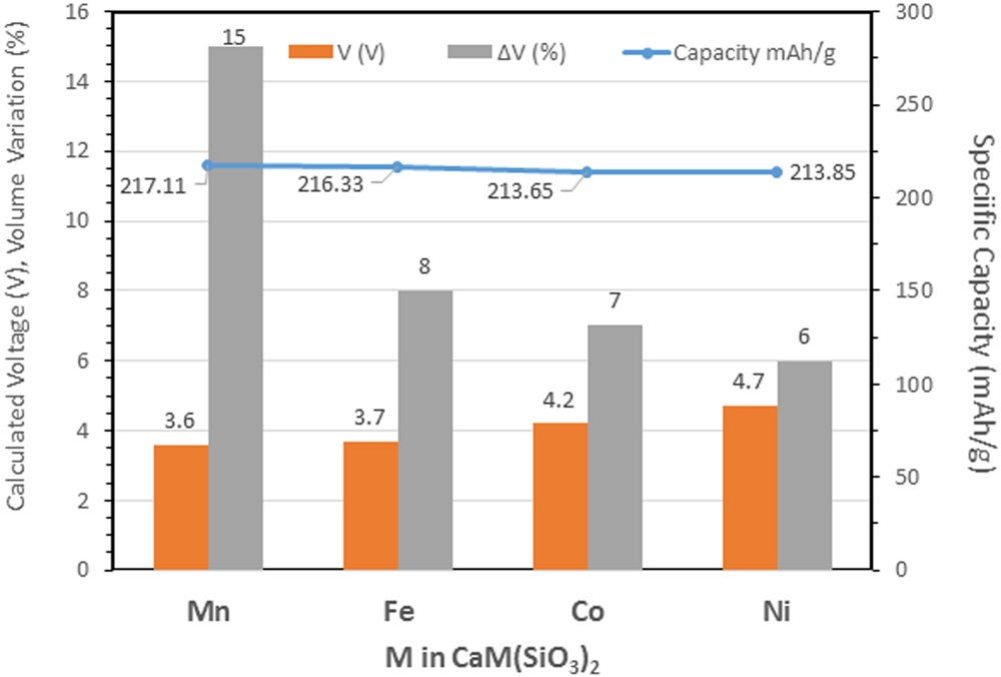
Calculated average voltage, volume variations and specific capacity associated within the pyroxene group. The volume variation is defined as ((V_initial_ − V_deintercalated_)/V_initial_) * 100.

Figure 4 compares the theoretical specific capacity, calculated volume variation, and intercalation voltage for various transition metals. Theoretical specific energies on the order of 800 mWh/g are expected for the pyroxene group. In addition, the deintercalation reaction cause limited changes in the crystal structure of Fe, Co and Ni pyroxenes (see Table 1 in S.I.), although sustainability and cost advocate the utilization of Mn.

Ca mobility in the pyroxene structure has been evaluated for CaMn(SiO_3_)_2_ using a Ca_7_Mn_8_Si_16_O_48_ supercell. The Ca ions in the rows of edge-sharing polyhedra along the *c*-axis are 5.3 Å apart. In the simplest hopping mechanism, a diffusing Ca ion can jump to a neighbouring vacant site by two different pathways: (i) across the common edge (purple arrow in Fig. 3c) and, (ii) across a triangular face via the occupation of a hexa-coordinated intermediate site (orange arrow, in Fig. 3c). The calculated energy barriers are, respectively, 4.6 eV and 6.8 eV. These large energy barriers are indicative of hindered Ca mobility. Figure 3d shows the energy landscapes associate to the hopping mechanism. The large energy barrier at the saddle point results from the very short cation-cation distances, this is to say, large electrostatic repulsions. In the intermediate site of path (i) the Ca^2+^-Si^4+^ distance is of only 2.56 Å, to be compared with the initial Ca^2+^-Si^4+^ distance of 3.12 Å. In path (ii) the intermediate hexa-coordinate site shares a face with the neighbouring Mn polyhedra (distance Ca^2+^-Mn^3+^  = 2.54 Å), hence, a diffusing Ca ion occupying this site will suffer strong electrostatic repulsion.

Cation diffusion is primarily determined by the crystal structure. Previous computational investigations show that for a given family of compounds such as LiMPO_4_, CaM_2_O_4_, MgM_2_S_4_ and so forth, the energy barrier expand in about 0.3 eV with the nature of the transition metal ion (M)^2,4,20,35^. Note that a 60 meV increase (decrease) in the migration energy corresponds to an order of magnitude decrease (increase) in diffusion coefficient^3^. Since the calculated energy barrier of CaMn(SiO_3_)_2_ is 4.6 eV, substitution of other transition metal for Mn is not expected to lower the energy barrier for Ca diffusion in pyroxenes below the acceptable threshold of 0.625 eV for nanosized particles to operate at C/2 rate.

### Garnet group

**Figure 5:**
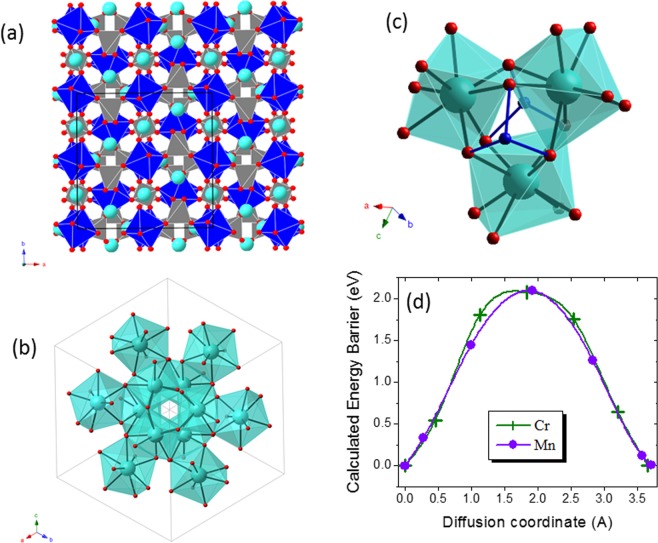
Analysis of garnet minerals. (**a**) The garnet structure along the [001] direction (**b**) Unit cell of the garnet structure showing the interconnected Ca sites forming a network for Ca diffusion. (**c**) Detail of the garnet structure showing three edge-sharing Ca dodecahedra. (**d**) Calculated energy barriers for Ca diffusion in garnet Ca_3_M_2_(SiO_4_)_3_ for M = Cr and Mn. Color code: Ca cyan, Cr/Mn blue, O red.

The garnet group of rock-forming minerals can be defined as a multicomponent substitutional solid solution series of silicates with the general formula X_3_Y_2_Si_3_O_12_, where X is a divalent cation (Mg, Fe, Mn, and Ca, in the most common species) in 8-fold coordination and Y is a trivalent cation (Al, Fe, and Cr, in the most common species) in 6-fold coordination^36^. The structure of these silicate garnets (Fig. 5a) consists of isolated Si tetrahedra (in grey) sharing corners with the Y octahedra (in blue) to form chains along each of the *a* axes of a cubic cell. The spaces between chains are occupied by the X cations (in cyan) which are coordinated by eight oxygen atoms forming a triangular dodecahedron (i.e. distorted cube). Each dodecahedron is linked to other three forming a 3D network of sites in the structure (see Fig. 5b). In addition to these silicate garnets, the garnet supergroup^37^ also includes minerals other than silicates and even those in which the anion is not oxygen (mainly OH^−^ groups or, more rarely, F^−^). All these minerals, however, are isostructural with the most common rock-forming silicate garnets described above.

In the context of rechargeable batteries technologies it is important to note that some Li-bearing synthetic garnet-type oxides have received much attention in the last years^38–41^. In some cases, Li is in tetrahedral or octahedral coordination, but in other cases Li occupies additional crystallographic sites not available in the “standard” garnet structure^37,41^. Some of these “Li-stuffed” garnet-type oxides do not have cubic symmetry^41^.

Regarding Ca-batteries, some garnets might be interesting as they contain transition elements in the octahedral and/or tetrahedral sites (Table 3 in S.I.), including both silicate garnets (of the schorlomite and garnet, s.s. groups) and oxide garnets (of the bitikleite group). Due to its relatively large ionic radius, Ca^2+^ is in the 8-fold coordination X site. Among the Ca-garnets, we have chosen uvarovite Ca_3_Cr_2_(SiO_4_)_3_ for which Ca deintercalation implies Cr^3+^ oxidation. For the Cr^3+^/Cr^6+^ redox couple, the theoretical capacity is 321 mAh/g and the calculated average voltage 4.8 V. However, the instability of current electrolytes for Ca batteries beyond 4 V^7^ limits the practical deliverable specific capacity of uvarovite.

The garnet structure possesses three-dimensional pathways for Ca diffusion; Fig. 5c shows a detail of three interconnected Ca sites. Figure 5d provides the calculated energy landscape for Ca ions hopping from one Ca site to the nearest one. A diffusing Ca ion must move from the initial site across a quadrangular face to fit in a trigonal prismatic (TP) site at the transition state. The calculated Ca-O distances in the quadrangular face and in the TP site range from 2.14 to 2.37 Å and from 2.13 to 2.51 Å, respectively. Having in mind the ionic radii, r (Ca^2+^) = 1.12 Ằ and r(O^2−^) = 1.4 Ằ, the Ca^2+^ ion seems too large for the size of the channel. Regarding the cationic repulsions, although the TP site shares a face with the octahedral chromium, the Ca-Cr^3+^ distance is 2.8 Å. This means that the cationic repulsion is lower than in the pyroxene structure, in agreement with its lower energy barrier of 2.07 eV (Fig. 5). However, this barrier is still too high for Ca mobility.

For the sake of completeness, we have calculated the energy barrier for migration in the garnet Ca_3_Mn_2_(SiO_4_)_3_ (theoretical capacity 105 mAh/g for the Mn^3+^/Mn^4+^ redox couple). Compared to Cr^3+^, the slightly larger and less polarizing High Spin-Mn^3+^ (r (Mn^3+^)_VI_ = 0.645 Ằ, r (Cr^3+^)_VI_ = 0.615 Ằ)^42^ may diminish the electrostatic repulsion and expand the crystal structure to facilitate Ca diffusion (see Table 1 in S.I. for cell volume). Yet, the calculated energy is 2.09 eV. This corroborates the aforementioned observation of a subtle energy barrier variation with the nature of the transition metal.

### Double carbonates

**Figure 6:**
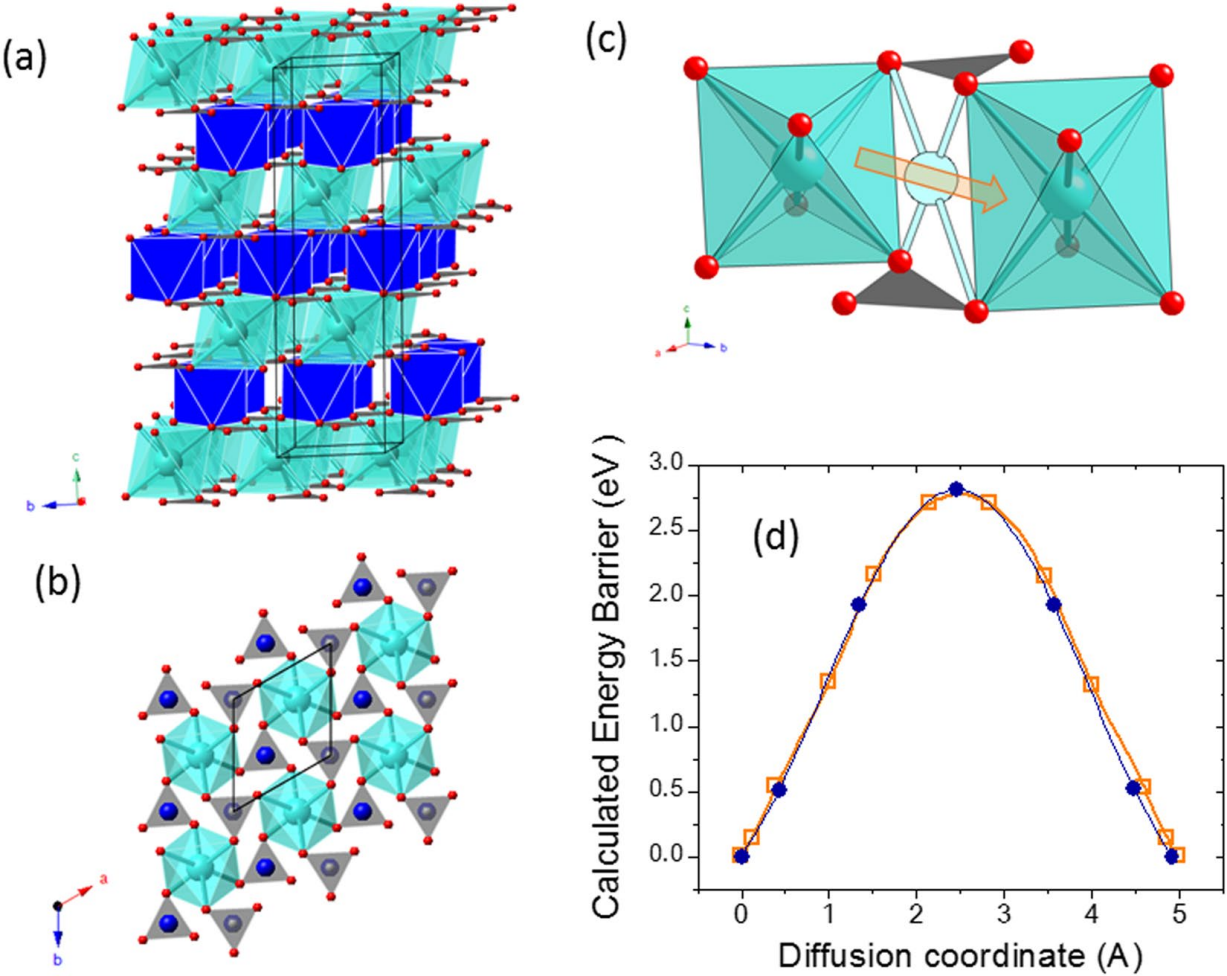
Analysis of double carbonates. (**a**) Crystal structure of CaMn(CO_3_)_2_ where M and Ca are ordered in alternate planes along the *c* axis of the hexagonal cell. (**b**) View of the Ca distribution in the *ab* plane. (**c**) Two neighbouring Ca sites in CaMn(CO_3_)_2_. The arrow indicates the pathway for Ca diffusion and the intermediate four-fold coordinated site. (**d**) Calculated energy barriers for Ca diffusion in kutnahorite with 5 and 10 intermediate images. Color code: Ca cyan, Mn blue, C grey, O red.

Natural carbonates with two cations (Ca + Mg/Fe/Mn/Zn) share a common structure type, usually defined as the dolomite (CaMg(CO_3_)_2_) structure. The dolomite structure may be considered to derive from that of calcite which, in turn, could be described as a derivative from the halite structure. Thus, compared to halite, in the calcite structure chlorine anions are substituted by the carbonate (CO_3_)^2−^ anionic groups and Na^+^ cations by Ca^2+^. The (CO_3_)^2−^ anionic groups form triangles in which each carbon is covalently bonded to three oxygens. Substitution of nearly-spherical chlorine anions by these planar groups results in a symmetry change from cubic to rhombohedral. This can be viewed as a shortening of the cubic cell along one of its largest diagonals (the 3-fold axis). Triangular (CO_3_)^2−^ groups are parallel and lie in horizontal layers perpendicular to this axis that becomes the *c*-axis; carbonate groups in adjacent layers, however, point in opposite directions. In the calcite structure, there are layers of (CO_3_)^2−^ groups and layers of Ca^2+^ cations which occupy 6-fold sites between the layers of the anionic groups. Compared to calcite structure, that of double carbonates shows an ordered arrangement of cations (Fig. 6a), because the structure must accommodate distinctly different-sized cations. In the idealized structure this is accomplished by having layers of Ca^2+^ alternating with layers of the smaller (Mg/Fe/Mn/Zn)^2+^ cations. Such an ordered cation arrangement results in a symmetry reduction respect to that of the calcite structure.

Double-cation carbonate minerals correspond to dolomite (CaMg(CO_3_)_2_), ankerite (CaFe(CO_3_)_2_), kutnahorite (CaMn(CO_3_)_2_) and minrecordite (CaZn(CO_3_)_2_). Isomorphic replacement among cations other than Ca^2+^ is common in these minerals, especially in ankerite (Ca(Fe,Mg)(CO_3_)_2_) and kutnahorite (Ca(Mn,Fe,Mg)(CO_3_)_2_). In addition, disordered structures at low temperatures have also been reported (e.g., in kutnahorite^43^). These authors suggest that the large ionic radius of Mn^2+^ relative to Mg^2+^ in dolomite leads to coupled distortion of the Ca and Mn octahedra that may result in a low ordering potential.

Ankerite and kutnahorite are interesting minerals as electrode for Ca batteries. The present DFT results indicate that CaMn(CO_3_)_2_ reunites excellent electrode characteristics: theoretical specific capacity of 250 mAh/g, calculated average voltage of 3.8 V and volume variation of 13%.

A Ca_11_Mn_12_C_24_O_72_ supercell has been used to study Ca diffusion in kutnahorite. As seen in Fig. 6b, the calcium ions occupy octahedral sites with oxygen at a distance of 2.36 Å. In the *ab* plane, each Ca has six equivalent Ca-neighbours at a distance of 4.89 Å. Although the Ca-O polyhedra are not interconnected, the arrow in Fig. 6c indicates the possible pathway for in-plane conductivity. In this path, the Ca ion moves from its initial site across a triangular face to fit in the intermediate distorted octahedral site and then reach the final Ca site. Nudged Elastic Band method (NEB) calculations have been performed for this path considering 5 and 10 intermediate images (Fig. 6d). The Ca ions at the saddle point resides in four-coordinated site only 2.51 Å apart from the central C atom of the nearest carbonate groups. Unfortunately, the calculated energy barrier is 2.8 eV, excluding the potential interest of double carbonates as electrode materials for Ca batteries.

## Discussion

Ca-bearing minerals combined with transition metals capable of being oxidized/reduced belong to various major mineral groups: olivine, amphibole, pyroxene, garnet and dolomite. Each mineral group has a chemical composition and crystal structure that determine the basic electrode characteristics, and eventually, the potential interest of these minerals as electrode materials for Ca batteries. Paradoxically, although one of the Li-cathode materials currently used in commercial batteries (LiFePO_4_) has the olivine structure, such a structure is not suitable for the Ca intercalation chemistry. For the other mineral groups, the chemical composition and the nature of the transition metal ions dictate the specific capacity and control the intercalation voltage. With the exception of the amphibole group, large specific capacities and voltages are predicted for the above mentioned Ca transition metal-bearing minerals.

Table 1 summarizes the calculated electrode characteristics for representative pyroxene, garnet and double carbonates minerals: average intercalation voltages, volume variation, energies barriers for Ca diffusion and band-gap for electronic conductivity. Band-gaps are extracted from the calculated density of states (see Fig. 1 in S.I.). These minerals display high theoretical energy densities with predicted moderate structural modifications. Their low electrical conductivity (electronic and ionic) is the main concern in terms of applications, as the electrical conductivity will determine the power and rate capability of the Ca battery. Particle size minimization (nanosizing) and intimate carbon coating is a strategy to improve the electronic conductivity, a typical example being the insulating LiFePO_4_ material (band-gap 3.5 eV)^20,44–47^. Nanostructuring is also a common route to mitigate poor ionic diffusion in electrodes^48–50^. Ceder *et al*. have estimated the relationship between the migration energy barrier and the maximum particle size permitting reasonable diffusivity in the context of battery performance^2,3^. For a given (dis)charge time, the cathode particle size will determine a maximum tolerable energy barrier for Ca migration. According to reference^2^, for a low rate performance (large (dis)charging time of 10 h) the energy barrier can be at most ∼0.6 meV when using a micron-sized particle and ∼0.9 meV in a nanosized particle (at 298 K). While these values demonstrate the potential importance of nanosizing to enhance rate capability, they also indicate that materials with very low intrinsic Ca diffusivity (energy barriers exceeding 1 eV) are not viable as electrodes, even at the nanoscale. That is the case of the investigated minerals, whose energy barriers exceeding 2 eV exclude practical applications.

**Table 1:** Computed properties (voltage, volume variation and energy barrier for Ca diffusion) of Ca-bearing transition metal minerals.

GROUP	Pyroxene	Garnet	Dolomite
COMPOSITION	CaMn(SiO_3_)_2_	Ca_3_Cr_2_(SiO_4_)_3_	CaMn(CO_3_)_2_
REDOX COUPLE	Mn^2+^/Mn^4+^	Cr^3+^/Cr^6+^	Mn^2+^/Mn^4+^
CAPACITY (mAh/g)	217	321	250
AVERAGE VOLTAGE (V)	3.6	4.8	3.8
ENERGY (mWh/g)	780	1540	950
VOLUME INCREMENT (%)	15	14	13
DIFFUSSION BARRIER (eV)	4.6	2.1	2.8
BAND GAP (eV)	3.4	3	3.8

The theoretical specific capacity for the corresponding redox couple is indicated.

Factors controlling Ca diffusion depend on crystal structure. The garnet, pyroxene and dolomite mineral groups possess suitable Ca migration pathways within the structure, but the local topology hampers Ca mobility. The garnet structure is well known for fast Li ion conductors, yet we found the pathways are too narrow for the large Ca ion. A general observation is the strong electrostatic interactions between the diffusing Ca^2+^ and the other cations (transition metal and central atom of the polyanionic groups) in the lattice. Generally speaking, the calculated energy barriers for Ca diffusion in minerals are larger than those reported in transition metal oxides^21,22,51^. A possible reason is the higher concentration of cations in these polyanionic structures, arising from the transition metals and the silicate/carbonate groups. Under this assumption, not only existing minerals, but also synthetic materials that emulate their crystal structures are not appealing as electrode materials for Ca batteries.

Regardless the usefulness of Ca-bearing minerals as positive electrode for Ca batteries, we should underline the importance of the abundant sulfate and carbonate mineral groups as the primary source for the Ca metal anode, which in the end sustain the interest of this technology, cheaper than that based on the Li-ion. As for the cathode side, so far TiS_2_ appears as a sustainable option (voltage ~1.8 V, specific capacity 240 mAh/g, energy barrier 0.7 eV)^8^, and an intensive DFT scrutiny is underway to identify oxides based on abundant transition metal ions.

## Methods

The calculations have been performed using the ab-initio total-energy and molecular dynamics program VASP (Vienna ab-initio simulation program) developed at the Universität Wien^52^. Total energy calculations based on DFT were performed within the General Gradient Approximation (GGA), with the exchange and correlation functional form developed by Perdew, Burke, and Ernzerhof (PBE)^53^. The interaction of core electrons with the nuclei is described by the Projector Augmented Wave (PAW) method^54^. The energy cut off for the plane wave basis set was kept fix at a constant value of 600 eV throughout the calculations. The integration in the Brillouin zone is done on an appropriate set of k-points determined by the Monkhorts-Pack scheme. A convergence of the total energy close to 10 meV per formula unit is achieved with such parameters. Spin polarized calculations were performed in all cases. Previous computational works demonstrate that accurate intercalation voltage prediction for polyoxoanionic compounds (silicates, phosphate, vanadates and so forth) requires introducing a Hubbard correction term in the calculation^55–57^. In this work, the total energies of the GGA-optimized compounds have been calculated using the GGA + *U* method, following the simplified rotationally invariant form of Dudarev *et al*.^58^ with *U* effective values of 3.7 eV (Cr), 4 eV (Mn), 5.3 eV (Fe), 3 eV (Co) and 6.9 eV (Ni) for the *d*-states of transition metal ions. The crystal models for the deintercalated phases were constructed removing all the Ca ions from the optimized structure of the initial minerals (CaMn(SiO_3_)_2_^59^, Ca_3_Cr_2_(SiO_4_)_3_^60^, Ca_3_Mn_2_(SiO_4_)_3_^61^, CaMn(CO_3_)_2_^43^). All crystal structures were fully relaxed (atomic positions, cell parameters and volume). The final energies of the optimized geometries were recalculated so as to correct the changes in the basis set of wave functions during relaxation.

Ca^2+^ mobility in selected minerals was investigated using the NEB method as implemented in VASP. NEB calculations have been carried out at the dilute limit x ~ 1, that is, the migration of a single vacancy in an otherwise fully inserted structure. To attain the dilute limit, we used superstructures of the unit cell that guarantees a minimum interaction between defects. Constant volume calculations were performed within the GGA approximation for five intermediate images initialized by linear interpolation between the two fully relaxed end points.

## Supplementary information

Analysis of minerals as cathode materials for Ca-based rechargeable batteries

## Data Availability

The data that support the findings of this study are available from the corresponding authors upon reasonable request.
