# Supplementary material for: Analysis of Minerals as Electrode Materials for Ca-based Rechargeable Batteries
Source: Sci Rep. 2019 Jul 4;9:9644. doi: 10.1038/s41598-019-46002-4 (PMC6609692; doi:10.1038/s41598-019-46002-4)
Supplement: Supplementary file 1 — Analysis of minerals as cathode materials for Ca-based rechargeable batteries [file 41598_2019_46002_MOESM1_ESM.pdf]

Supplementary Information

Analysis of Minerals as Electrode Materials for Ca-based Rechargeable Batteries

A. Torres[1], F. J. Luque[2,3], J. Tortajada[4] and M. E. Arroyo-de Dompablo[1*]

1 Departamento de Química Inorgánica, Universidad Complutense de Madrid, 28040 Madrid (Spain).

2 Dpto. de Mineralogía y Petrología, Facultad de Geología, Universidad Complutense de Madrid, 28040 Madrid (Spain).

3 Dpto. Geomateriales, Instituto de Geociencias IGEO (CSIC, UCM), 28040 Madrid (Spain).

4 Departamento de Química Física, Universidad Complutense de Madrid, 28040 Madrid (Spain).

*Corresponding author: e.arroyo@quim.ucm.es

Table 1. Calculated lattice parameters and comparison with available experimental values

COMPOSITION	*a* (Å)	*b* (Å)	*c* (Å)	β / γ (º)	Volume (Å^3)
**PYROXENE**					
$CaMn(SiO_3)_2$	10.058	9.242	5.343	105.299	479.19
Experimental[1]	9.9227(3)	9.1416(3)	5.2745(2)	105.087(3)	461.96
$Mn(SiO_3)_2$	9.502	8.559	5.327	110.443	406.02
$CaFe(SiO_3)_2$	10.035	9.043	5.329	105.183	466.74
Experimental[2]	9.845(1)	9.024(1)	5.245(1)	104.74(1)	450.64
$Fe(SiO_3)_2$	9.678	8.738	5.352	109.084	427.74
$CaCo(SiO_3)_2$	9.930	9.034	5.316	105.910	458.66
Experimental[3]	9.7970(4)	8.9577(4)	5.2445(2)	105.513(3)	443.48
$Co(SiO_3)_2$	9.671	8.710	5.349	109.430	424.90
$CaNi(SiO_3)_2$	9.864	8.959	5.294	106.231	449.22
Experimental[4]	9.7359(4)	8.8932(4)	5.2284(3)	105.803(3)	435.52
$Ni(SiO_3)_2$	9.891	8.708	5.285	111.620	423.25
**GARNET**					
$Ca_3Cr_2(SiO_4)_3$	12.153	12.153	12.153		1795.27
Experimental[5]	11.950(3)	11.95	11.95		1706.49
$Cr_2(SiO_4)_3$	11.576	11.576	11.576		1551.44
$Ca_3Mn_2(SiO_4)_3$	12.228	12.228	12.228		1828.55
Experimental[6]	12.07	12.07	12.07		1758.42
**DOLOMITE**					
$CaMn(CO_3)_2$	4.936	4.936	16.544	120	349.14
Experimental[7]	4.894	4.894	16.500	120	342.25
$Mn(CO_3)_2$	4.668	4.668	16.142	120	304.61

Table 2. Cation occupancy of structural sites in pyroxene minerals[8]

Group	***X cations (M2)***	***Y cations (M1)***	***Z cations***	***Symmetry***
Magnesium-iron	Mg^{2+}, Fe^{2+}	Mg^{2+}, Fe^{2+}	Si^{4+}	Orthorhombic
Calcium	Ca^{2+}, (Mg^{2+}, Fe^{2+})	Mg^{2+}, Fe^{2+}, Al^{3+}	Si^{4+}, Al^{3+}	Monoclinic
Calcium-sodium	Ca^{2+}, Na^{+}	Mg^{2+}, Fe^{2+}, Al^{3+}, Fe^{3+}	Si^{4+}	
Sodium	Na^{+}	Al^{3+}, Fe^{3+}	Si^{4+}	
Lithium	Li^{+}	Al^{3+}	Si^{4+}	

Table 3. Ca-bearing garnets (adapted from [9] and AMCSD data base)

Group	***Class***	***Mineral***
BITIKLEITE	Oxide	Bitikleite Ca_3 $Sb^{5+}Sn^{4+}$ Al_3 O_{12}
		Usturite Ca_3 $Sb^{5+}Zr$ Fe^{3+}_3 O_{12}
		Dzhuluite Ca_3 $Sb^{5+}Sn^{4+}$ Fe^{3+}_3 O_{12}
		Elbrusite Ca_3 $U^{6+}_{0.5}Zr_{1.5}$ Fe^{3+}_3 O_{12}
SCHORLOMITE	Silicate	Schorlomite Ca_3 Ti_2 $SiFe^{3+}_2$ O_{12}
		Irinarassite Ca_3 Sn^{4+}_2 $SiAl_2$ O_{12}
		Toturite Ca_3 Sn^{4+}_2 $SiFe^{3+}_2$ O_{12}
GARNET	Silicate	Andradite Ca_3 Fe^{3+}_2 Si_3 O_{12}
		Uvarovite Ca_3 Cr^{3+}_2 Si_3 O_{12}
		Goldmanite Ca_3 V^{3+}_2 Si_3 O_{12}
		Morimotoite Ca_3 $TiFe^{2+}$ Si_3 O_{12}

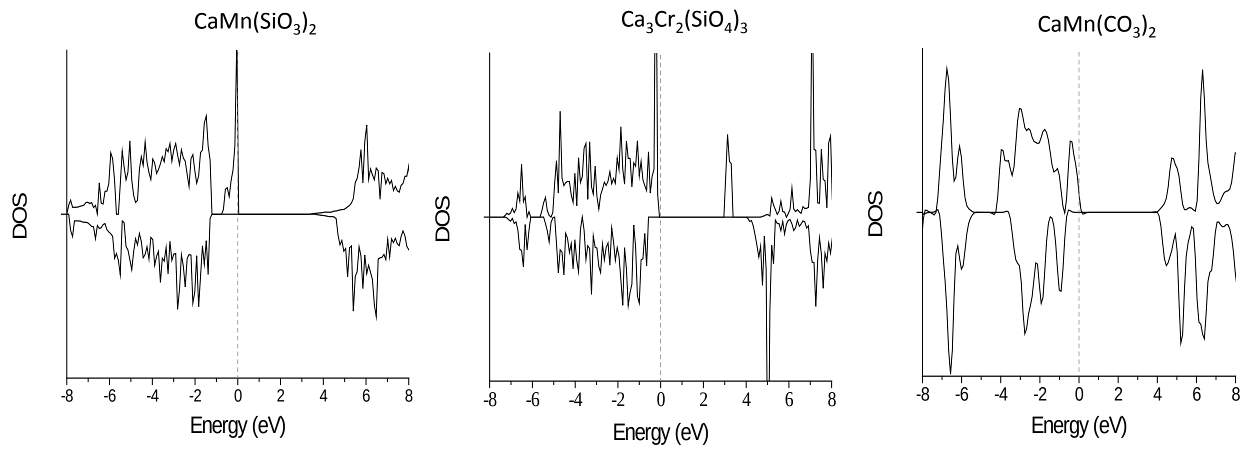

Figure 1. Calculated density of states (DOS) for representative pyroxene, garnet and dolomite-like minerals. The Fermi level is set as the zero of energy.

References

1 Nestola, F., Ballaran, T. B., Angel, R. J., Zhao, J. & Ohashi, H. High-pressure behavior of Ca/Na clinopyroxenes: The effect of divalent and trivalent 3d-transition elements. *American Mineralogist* **95**, 832-838 (2010).

2 Cameron, M., Sueno, S., Prewitt, C. T. & Papike, J. J. High-temperature crystal chemistry of acmite, diopside, hedenbergite, jadeite, spodumene, and ureyite. *American Mineralogist* **58**, 594-618 (1973).

3 Durand, G., Vilminot, S., Rabu, P., Derory, A. & Lambour, J. P. Synthesis, structure, and magnetic properties of $CaMSi_2O_6$ (M = Co, Ni) compounds and their solid solutions. *Journal of Solid State Chemistry* **124**, 374-380 (1996).

4 Raudsepp, M., Hawthorne, F. C. & Turnock, A. C. Crystal chemistry of synthetic pyroxenes on the join $CaNiSi_2O_6$-$CaMgSi_2O_6$ (diopside): A Rietveld refinement study. *American Mineralogist* **75**, 1274-1281 (1990).

5 Menzer, G. The crystal structure of garnets. *Zeitschrift Fur Kristallographie* **69**, 300-396 (1928).

6 Novak, G. A. & Gibbs, G. V. The crystal chemistry of the silicate garnets. *American Mineralogist* **56**, 791-823 (1971).

7 Peacor, D. R., Essene, E. J. & Gaines, A. M. Petrologic and crystal-chemical implications of cation order-disorder in kutnahorite $CaMn(CO_3)_2$. *American Mineralogist* **72**, 319-328 (1987).

8 Cameron, M. & Papike, J. J. Structural and chemical variations in pyroxenes. *American Mineralogist* **66**, 1-50 (1981).

9 Grew, E. S. *et al.* Nomenclature of the garnet supergroup. *American Mineralogist* **98**, 785-811 (2013).
